# The occurrence of postpartum post-traumatic stress disorder among women in Johannesburg metropolitan clinics

**DOI:** 10.4102/sajpsychiatry.v32i0.2678

**Published:** 2026-08-10

**Authors:** Botho P. Pheto-Malikongwa, Melitah Motlhale, Reyanta Bridgmohun, Danie Hoffman, Anthony Olashore

**Affiliations:** 1 Department of Psychiatry, Faculty of Medicine, University of Botswana, Gaborone, Botswana; 2 Department of Epidemiology and Surveillance, National Health Laboratory Service, National Institute for Occupational Health, Johannesburg, South Africa; 3 Department of Psychiatry, Faculty of Medicine, University of KwaZulu-Natal, Durban, South Africa; 4 Department of Psychiatry, Faculty of Health Science, University of the Witwatersrand, Johannesburg, South Africa

**Keywords:** postpartum, post-traumatic stress disorder, childbirth, maternal mental health, traumatic birth, mental health

## Abstract

**Background:**

Childbirth has been associated with post-traumatic stress disorder (PTSD) in women, affecting mother–child bonding, foetal growth, cognitive development and marital relationships. However, there is a dearth of literature on postpartum PTSD in Africa.

**Aim:**

The study aimed to investigate the occurrence of postpartum PTSD among women.

**Setting:**

The study was conducted within Johannesburg metropolitan clinics.

**Methods:**

The study included 88 women attending postnatal clinics in the metropolitan area. A questionnaire was used to collect data on their characteristics, obstetric and neonatal factors and perceived social support. The City Birth Trauma Scale was used to screen for postpartum PTSD symptoms. Data were analysed using R software with a significance level of 0.05.

**Results:**

Almost half of the participants (48.9%) perceived their most recent childbirth as a traumatic event. One participant (1.1%; 95% CI: 0.0003–0.06) endorsed all symptoms required to diagnose PTSD. Endorsing childbirth as a traumatic event was significantly associated with reporting intrusion and/or re-experiencing symptoms (OR = 3.7) and avoidance symptoms (OR = 3.3). There was no association between the mode of childbirth and PTSD symptom scores. There is no association between the presence or absence of neonatal complications and PTSD symptom scores.

**Conclusion:**

Although the occurrence was relatively low, our findings highlight the importance of routine screening and early intervention in maternal health services.

**Contribution:**

There is need for further research to inform culturally relevant prevention and treatment strategies.

## Introduction

Conceiving and giving birth is a significant and special event that many women cherish and regard as a happy and enriching experience^1,2^; however, for some women, it can lead to psychological distress, which can trigger mental health disorders, including postpartum stress disorder.^1,2^ An increasing body of evidence suggests that the experience of childbirth may be linked to the development of postpartum post-traumatic stress disorder (PTSD) and that it is more common than previously believed.^3,4^ Early research on PTSD was mainly associated with military veterans; it is only in recent years that PTSD following childbirth is being recognised and investigated.^5^ There has been debate as to whether childbirth should be regarded as a traumatic event, as it is not sudden or unexpected and voluntary.^6,7^ Post-traumatic stress disorder is classified as a trauma and stressor-related disorder in the Diagnostic and Statistical Manual of Mental Disorders (DSM) 5.^8^ It is characterised by symptom clusters such as re-experiencing, avoidance, negative cognitions and mood, as well as hyperarousal, following exposure to a traumatic event.^8^ A traumatic event involves personally experiencing or being exposed to actual or threatened death or serious injury to oneself or others. Studies have shown that the childbirth experience can result in the woman perceiving that her life or that of her child is in danger, thereby fulfilling this criterion.^4,9^ Research on postpartum mental health has largely focused on postpartum depression, and while many are familiar with postpartum depression and psychosis, there is a dearth of literature on the phenomenon of postpartum PTSD, particularly in Africa.^6,10^ A systematic review investigating psychological disorders in the pre- and postnatal period among women in Africa found that depression was the most commonly assessed disorder.^11^ The consequences of postpartum PTSD are varied, warrant attention and may be considered a public health concern as it may adversely affect mother–child bonding and impair foetal growth and cognitive development, as well as marital difficulties.^12,13^ The first descriptions of traumatic experiences associated with childbirth were documented in case study.^14^ These authors documented women who experienced intrusive symptoms such as nightmares about giving birth, sometimes even years after the births.^1,14^ Over time, qualitative and quantitative studies reported women developing PTSD symptoms after childbirth, even after successful health outcomes.^10,14^ In 2000s, further research suggested that PTSD can occur as a result of childbirth after controlling for pre-existing psychiatric conditions before childbirth.^3,4^ Postpartum PTSD can be conceptualised in two ways. Firstly, it can occur as a result of the childbirth experience that triggers pre-existing PTSD. Secondly, it can indicate new-onset PTSD that is induced by childbirth itself.^12^ A stress-diathesis approach has been used to identify factors giving rise to postpartum PTSD; it is described as an interplay between pre-birth vulnerability factors, risk factors in birth and factors after birth.^15^ Factors that have been identified in the predictive model for women with traumatic childbirth at risk of developing postpartum PTSD include low social support and poor coping.^16^ There are variable estimates of the prevalence of postpartum PTSD, with more recent studies indicating rates ranging between 3.4% and 11%.^14^ The differences in rates may be attributable to several factors, including differences in the time of measurement of PTSD symptoms, different screening tools for PTSD, sample sizes and methodological approaches.^5,17^ The majority of studies were undertaken in Western and Middle Eastern countries.^6,7,18^ A systematic review reported a prevalence of 4.9% in women who did not have a history of pre-existing PTSD before childbirth. This systemic review of 36 articles included studies with high methodological ranking and controlled for PTSD symptoms before childbirth.^12^ The dearth of studies on postpartum PTSD in Africa is a matter of concern, particularly considering the potential implications for maternal health and well-being. Against this backdrop, a cross-sectional survey was conducted in Nigeria to determine the prevalence of postpartum PTSD.^19^ The survey involved a cohort of 876 women who were 6 weeks postpartum and were examined to determine the presence of postpartum PTSD. The study estimated a prevalence of 5.9% for postpartum PTSD, which suggests a significant incidence of this disorder among postpartum women in Nigeria.^19^ In 2024, a community based cross-sectional study conducted in south west Ethiopia found a 21.6% prevalence rate of postpartum PTSD from 624 participants. The identified risk factors included postpartum intimate partner violence, caesarean section delivery, instrumental delivery and maternal morbidity.^20^ Despite this, no study has been found that investigates the prevalence of PTSD in relation to childbirth in South Africa. This gap in the literature highlights the need for further research to be conducted in this area; hence, the current study aimed to investigate the occurrence of PTSD symptoms among women in Johannesburg metropolitan clinics at 6 weeks postpartum. The present study aims to build upon previous research in the field and provide a better understanding of the prevalence of postpartum PTSD in African contexts. Such research can help in the development of evidence-based interventions to improve the quality of care provided to women during childbirth.

## Research methods and design

### Study design and site

The study was a cross-sectional design and was conducted in postnatal clinics in the Johannesburg metropolitan area. The city is divided into seven regions (A-G), covering areas from Randburg, Sandton, Soweto, Roodepoort and Johannesburg inner city. Region D of the Johannesburg metropolitan area covers greater Soweto and its surrounding areas. The routine 6-week postnatal follow-up for women in Johannesburg is usually conducted at the district clinics and is independent of the place of delivery; hence, it involves mothers who delivered in hospitals and community obstetric units. Data were collected from three sites in Soweto, namely Mofolo Community Health Centre (CHC), Chiawelo and Lillian Ngoyi clinics, which form the largest centres serving the metropolitan area.

### Study sampling and inclusion criteria

The study employed a non-probability approach. The clinics were selected through facility-based convenience sampling and chosen for their large patient volumes, accessibility and wide representation. The required sample size was determined to be at least 87, using Fisher’s formula for a cross-sectional study,^21^ where *P* is the expected proportion who have the characteristic of interest (5.9%), and E is the margin of error (0.05). *n* is the sample size. Zα is a value from the normal distribution related to the significant level α. Zα = 1.96. Therefore, *n* = (1.96) ^2(0.059) (0.941) / (0.05) ^2 = 87. Inclusion criteria were postpartum women over the age of 18 years old and not more than 6 weeks postpartum. They were required to understand English.

### Study measures

The researcher designed a questionnaire that elicited the participants’ demographic characteristics and details on their most recent childbirth, including the place and mode of delivery. Additionally, the questionnaire sought information on neonatal characteristics, such as Apgar scores and neonatal complications. The City Birth Trauma Scale (CBTS) was used to screen for PTSD symptoms. It is a measure for PTSD in the postpartum period and was developed to measure PTSD according to the, DSM-5. It is a 29-item questionnaire and has been shown to have excellent reliability.^9^ The total score ranges from 0 to 60, with diagnostic criteria for PTSD specified from questions 1–22. It goes on to specify if there is PTSD with dissociative symptoms or PTSD with delayed onset in questions 23–25.^9^ Intrusion symptoms (criterion B of PTSD) were fulfilled if the women had a score of 1 or more on Q3–Q7 of the CBTS; likewise, avoidance symptoms were fulfilled if a woman had a score of 1 or more on Q8 and Q9. They fulfilled criterion D of PTSD if they scored 1 or more on Q10–16 and Q17–22 of the CBTS, respectively. The CBTS has yet to be validated in South Africa. However, it was selected for use in this study due to its adherence to the latest diagnostic criteria for PTSD, and permission was obtained from the scale’s developer to use it in this study.

### Study procedure

Data collection was undertaken from September 2020 to December 2020. The participants were women booked for their routine 6-week postpartum checkup and were all exactly 6 weeks post-delivery. With the assistance of the clinic register, all women attending the facility during the study period were screened against the predefined inclusion criteria. All those who met inclusion criteria were invited to participate in the study. Should any participant have experienced distress related to the nature of the study questionnaires, arrangements were in place for referral to a clinical psychologist.

### Data analysis

Statistical analyses were conducted in R software (R Core Team, 2024; Vienna, Austria. 4.00; www.R-project.org). The normal distribution of the data was checked using the Shapiro–Wilk test and Q-Q plots. Categorical data were analysed using Chi-squared contingency tests or Fisher’s exact tests (FET), for which interquartile and odds ratios are provided. Continuous variables were analysed using Mann–Whitney U (MWU) tests because the data were nonparametric. Tests were two-tailed, and model significance was set at 0.05. Data were reported as counts and percentages and median (interquartile range [IQR]), as appropriate, and are presented descriptively in charts, tables or text.

### Ethical considerations

Ethical clearance to conduct this study was obtained from the University of the Witwatersrand Human Research Ethics Committee (Ref. No. M200262). Permission was also obtained from the Johannesburg District Committee. The participants were offered information sheets and an informed consent document with details of the study and given an opportunity to ask questions. Only those who consented were included in the study.

## Results

### Sample characteristics

Data were collected from 88 out of 90 women from three Johannesburg metropolitan clinics, as two women declined to participate in the study. The socio-demographic and clinical variables recorded in the study population are presented in Table 1.

**TABLE 1:** Sample characteristics (*N* = 88).

Descriptions	Variables	*n*	%
Age (years)	< 35	72	81.8
	> 35	16	18.2
Highest level of education	< Matric	25	28.4
	Matric	53	60.2
	Tertiary	10	11.4
Employed	Yes	19	21.6
Relationship status	Single	76	86.4
	Married	12	13.6
Supportive partner (*n* = 87)	Yes	78	88.7
Supportive family (*n* = 87)	Yes	83	94.3
Previous children (*n* = 87)	1	27	30.7
	2	28	31.8
	3	20	22.7
	> 3	12	13.7
Planned pregnancy (*n* = 87)	Yes	35	39.7
Type of delivery	Caesarean section	28	31.8
	Vaginal delivery	60	68.2
Place of delivery	Clinic (MOU)	28	31.8
	Hospital	59	67.1
Complications (*n* = 87)	Yes	14	15.9
Use of analgesia during labour (*n* = 87)	Yes	40	45.4
Supportive nursing staff during labour (*n* = 87)	Yes	78	88.7

MOU, Midwife Obstetric Unit.

### Occurrence of post-traumatic stress disorder

Only one (1.1%; 95% CI: 0.0003–0.06) of the 88 participants in this study fulfilled the DSM 5 criteria for PTSD using CBTS.

### Socio-demographic characteristics of the study participants

Eighty-eight women participated in the study, the majority (81.8%) were below 35 years, 60.2% had a secondary education, while 86.4% were unmarried. In addition, 88.7% indicated that they had supportive partners.

### Obstetric and neonatal characteristics

Of the total study participants, 39.7% reported their most recent pregnancy was a planned one, 68.2 % delivered via normal vaginal delivery and 67.1% delivered in a hospital setting. As identified, 15.9% reported neonatal complications.

### Childbirth is endorsed as a traumatic event

**FIGURE 1:**
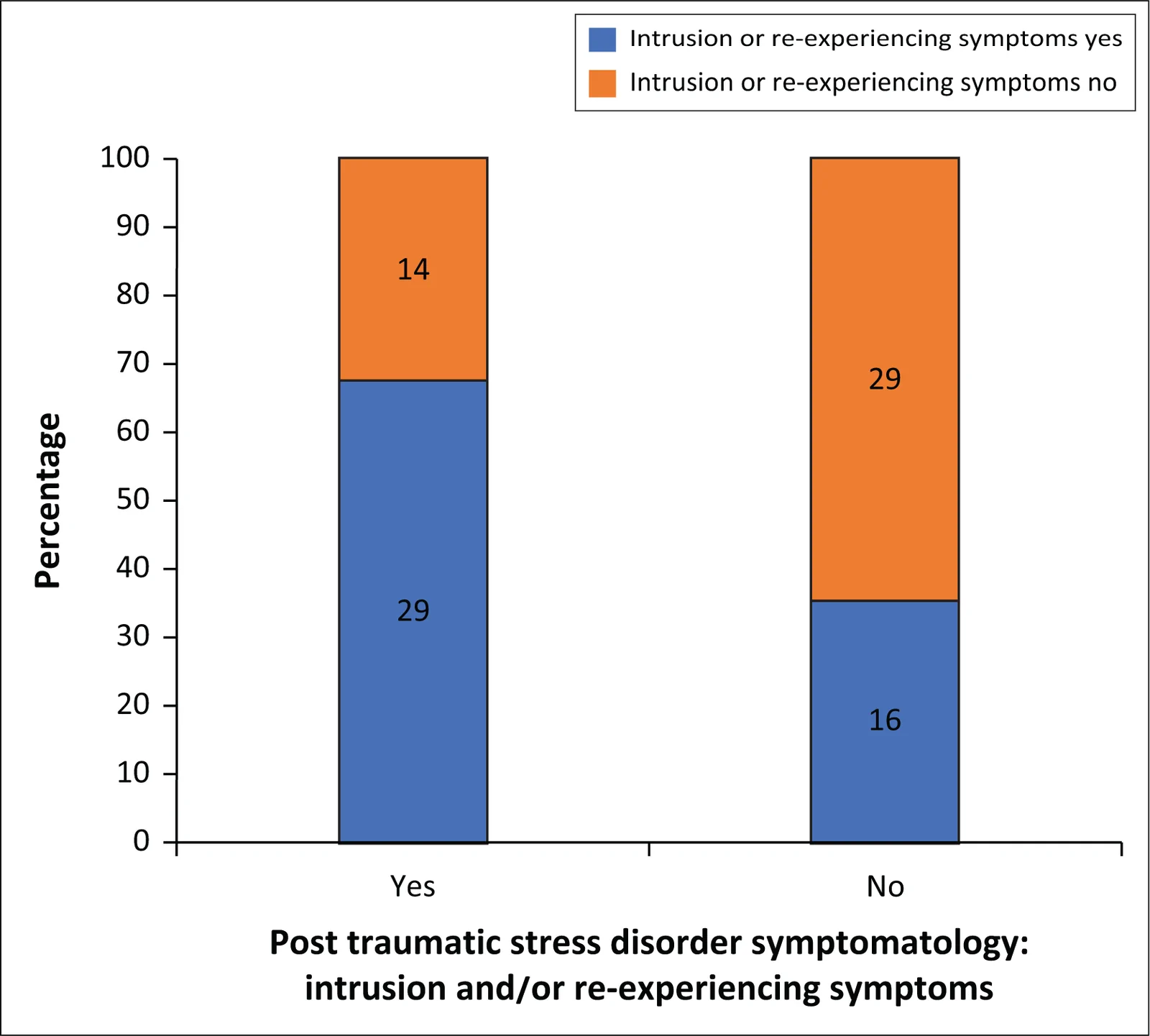
The relationship between viewing childbirth as a traumatic event and intrusion/re-experiencing symptoms.

**FIGURE 2:**
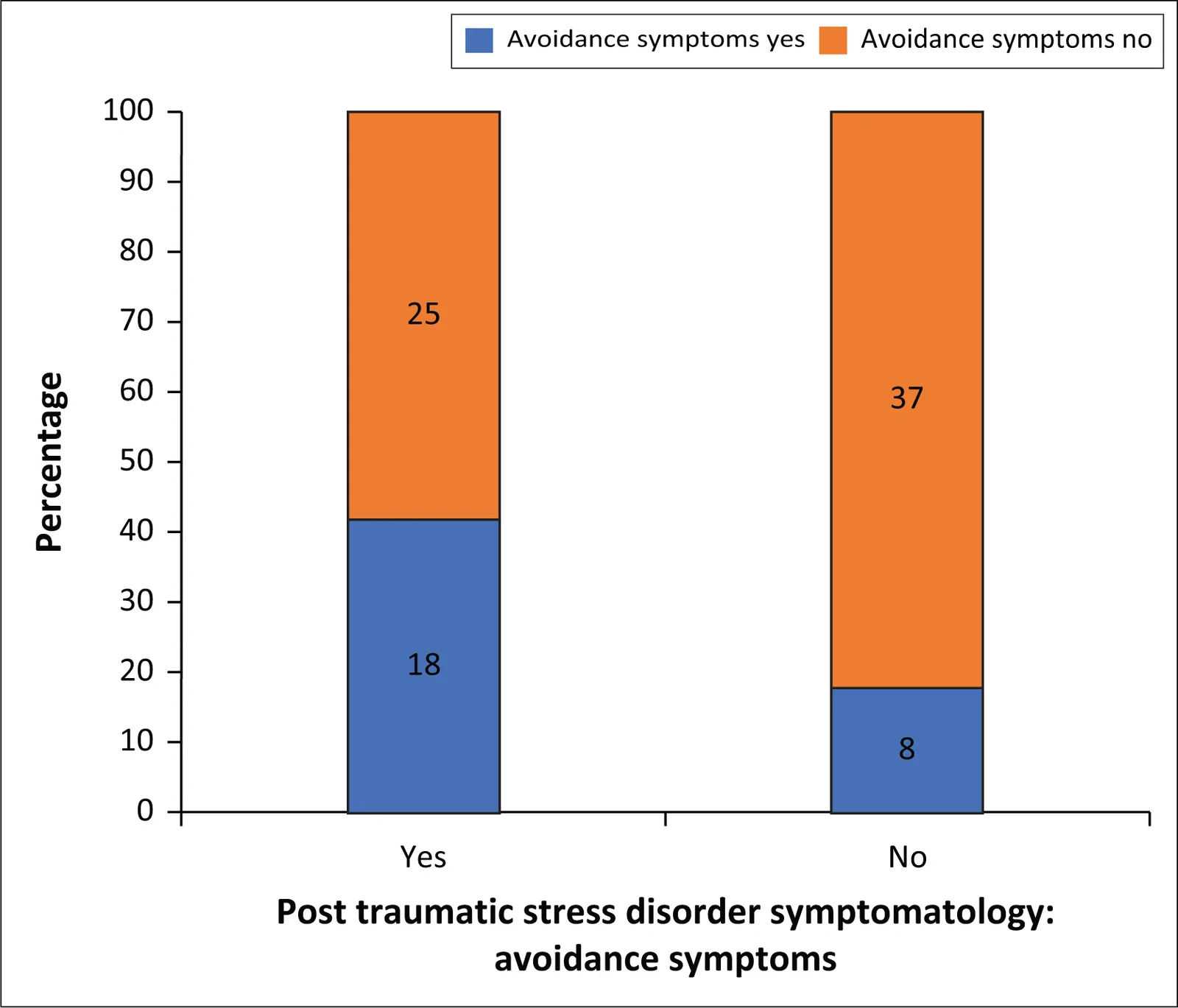
The relationship between viewing childbirth as a traumatic event and avoidance symptoms.

**FIGURE 3:**
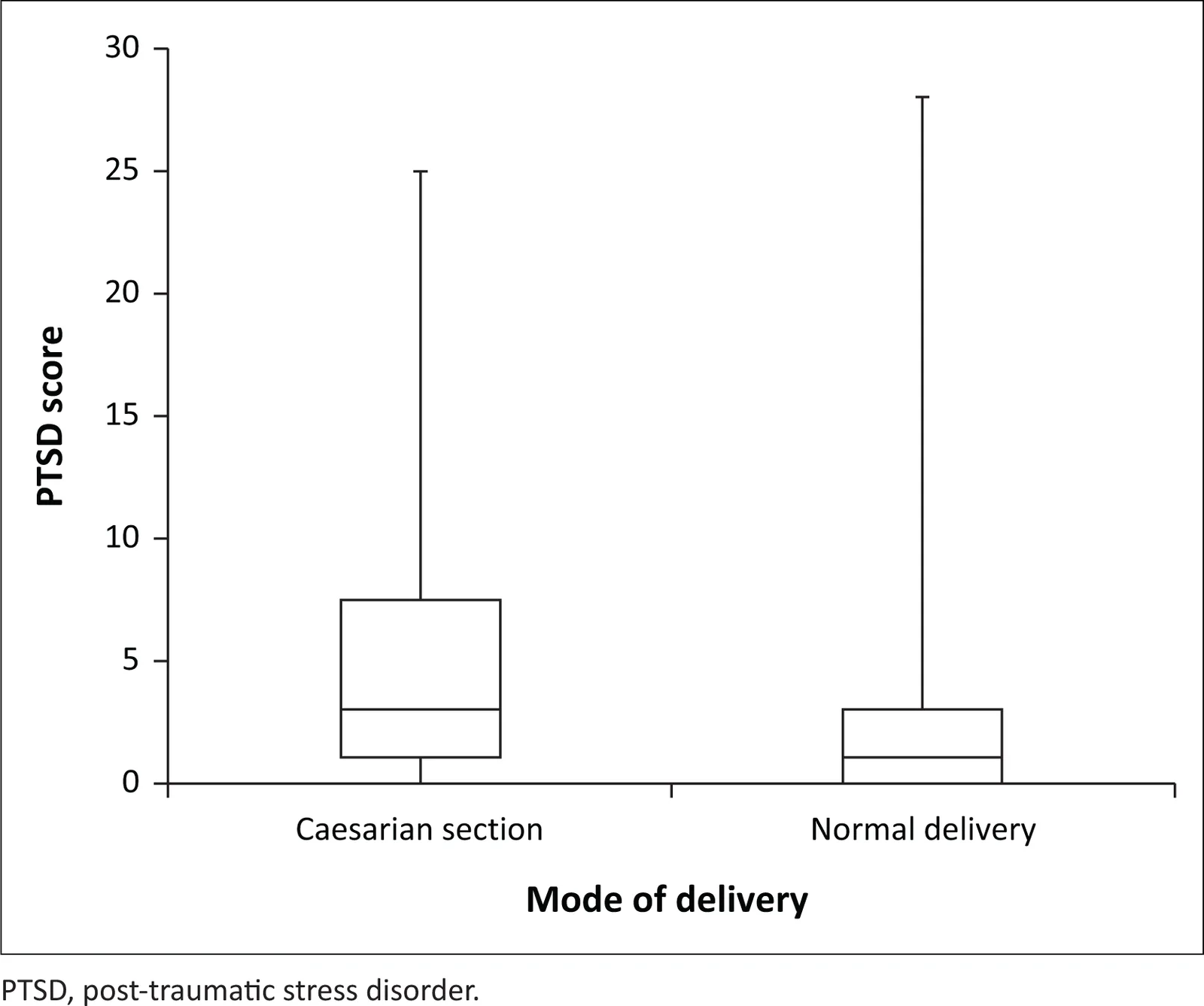
The difference in the median score of post-traumatic stress disorder symptoms between the modes of childbirth (i.e. caesarean and normal childbirth).

**FIGURE 4:**
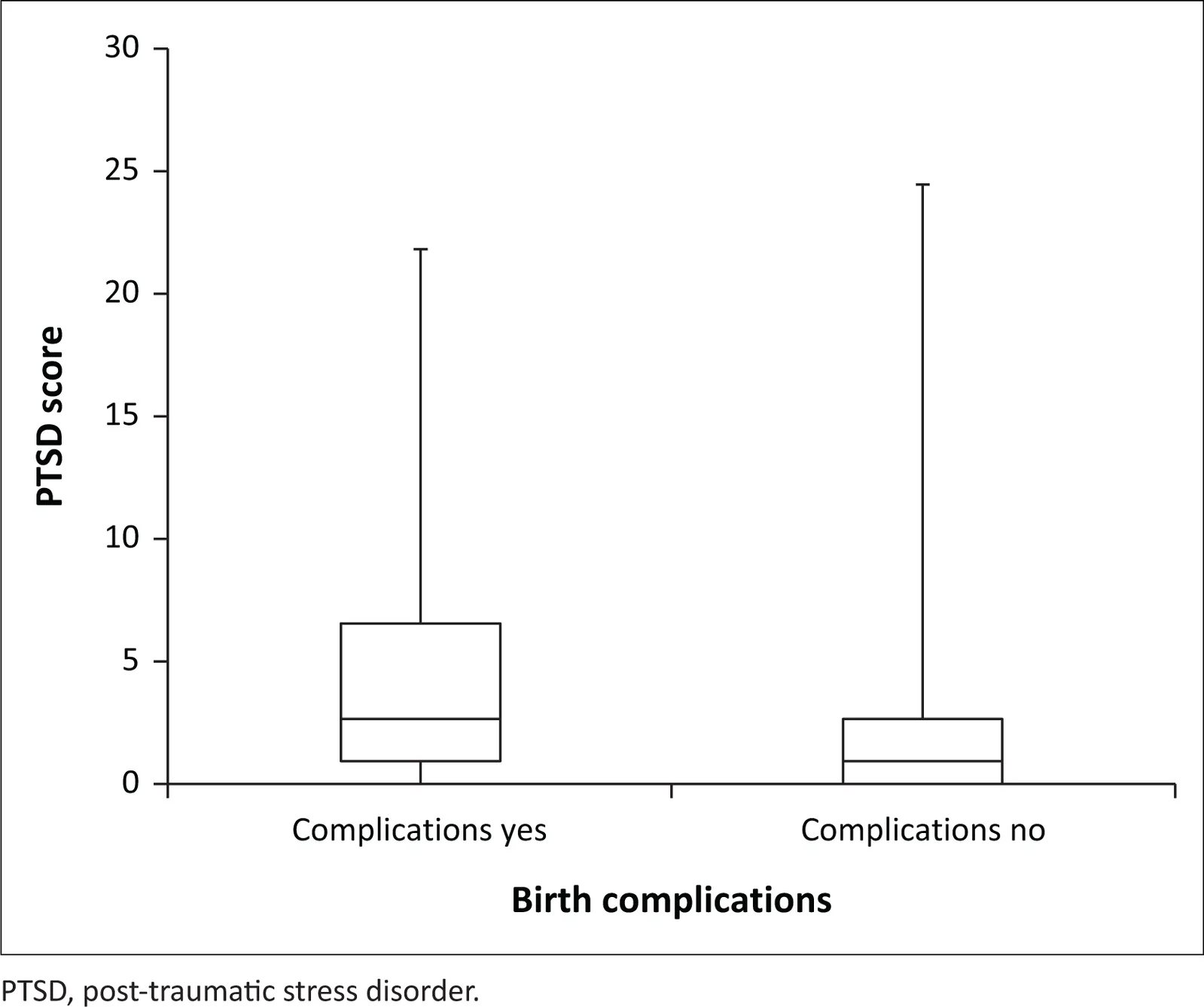
The difference in the median scores of post-traumatic stress disorder symptoms between the presence or absence of neonatal complications.

Nearly half of the women (*n* = 43, 48.9%) perceived their most recent childbirth as a traumatic event (criterion A of PTSD), believing either themselves or their baby would be seriously injured or would die (Q1 and/or Q2). The median (IQR) PTSD symptom score was median 2 and interquartile 3.6. Comparing the perceived traumatic event (childbirth) against PTSD clusters indicated that women who reported the birth as a traumatic event had higher PTSD scores (*n* = 43 median = 3, IQR = 1.75) than women who did not report the birth as a traumatic event (*n* = 45, median = 0, IQR = 0.3) (MWU = 603.5, *n* = 43, *n* = 45, *p* = 0.002). An analysis of the responses in the CBTS indicated that one woman fulfilled the full criteria for PTSD 6 weeks after birth. Of the 45 women who did not report childbirth as a traumatic event, some went on to report PTSD symptomatology (Table 2). Among the 43 women who perceived childbirth as a traumatic event, 29 (67%) also reported experiencing intrusion symptoms (FET = 0.003, 95% CI = 1.42–9.99) (Figure 1). In particular, those who reported childbirth as a traumatic event were 3.7 times (odds ratio) more likely to have intrusion or re-experiencing symptoms. Similarly, significantly more women who reported childbirth as a traumatic event also reported having avoidance symptoms (FET = 0.020, 95% CI = 1.15–10.17) (Figure 2). Specifically, they were 3.3 times more likely to have avoidance symptoms. Stressors had no significant influence on negative cognition or mood symptoms (FET = 0.276, 95% CI = 0.57–7.29) and hyperarousal symptoms (FET = 1.000, 95% CI = 0.25–6.98). There was no statistical difference in the median score of PTSD symptoms between the mode of childbirth (i.e. caesarean and normal childbirth) (MWU; *p* = 1.000) (Figure 3). There was no statistical difference in the median scores of PTSD symptom between the presence or absence of neonatal complications (MWU; *p* = 0.102) (Figure 4).

**TABLE 2:** Clusters of post-traumatic stress disorder symptomatology and frequency distribution.

DSM criteria for PTSD	Presence of PTSD symptoms	*n*	%
Believing mother or baby would be seriously injured or die	Yes	43	48.9
	No	45	51.1
One or more symptoms of intrusion or re-experiencing	Yes	29	67
	No	16	36
One or more symptoms of avoidance	Yes	18	42
	No	8	18
Two or more of negative mood and cognition	Yes	10	23
	No	6	13
Two or more symptoms of hyper vigilance	Yes	5	12
	No	4	9

DSM, Diagnostic and Statistical Manual of Mental Disorders; PTSD, post-traumatic stress disorder.

## Discussion

The study examined the occurrence of PTSD in a cohort of women 6 weeks postpartum. The results showed that 1.1% of women fulfilled the criteria for PTSD according to the CBTS at 6 weeks postpartum. In addition, a large portion of women (48.9%) perceived their most recent childbirth to be a psychologically traumatic event. This is similar to work done in Australia which found 45.5% of women are likely to report childbirth as being a traumatic experience.^4^ The results of the study have highlighted that women who did not perceive the experience of childbirth as a traumatic event subsequently experienced symptoms of post-traumatic stress, such as intrusion and avoidance. This may be attributed to a number of factors, including the potential challenge of comprehending the screening tool’s questions. Additionally, it could suggest that women may be hesitant to express negative emotions surrounding childbirth, which is often regarded as a joyous occasion by society. However, it may also be interpreted as an appropriate response to a significant life event like childbirth.^22^ The PTSD prevalence found in this study is lower than a prevalence rate of 4.9% reported in a meta-analysis in 2017; however, previous studies have reported similar rates.^22^ In the same vein, our rate is also notably lower compared to a similar study done in Nigeria, which estimated a prevalence rate of 5.9%.^19^ Although both studies examine postpartum PTSD in non-western settings at 6 weeks postpartum, there are several factors that may contribute to the differences in prevalence rates; these include differences in sample size and the different measures used. The study conducted in Nigeria used a clinically structured interview and psychiatric interview to diagnose PTSD and had a much larger sample. The present study used the CBTS, which is a recent measure based on DSM 5 criteria for PTSD, while the study in Nigeria used the Mini International neuropsychiatric interview for psychiatric disorders in DSM 4. The current study was unable to identify associated factors reliably; however, there may be differences between the two countries with regard to access to obstetric care and practices. Other factors may be differences in exposure to trauma in either country or different cultural contexts.^23^ Both studies did not control for pre-existing maternal PTSD; therefore, they are unable to identify if the PTSD symptoms endorsed are a result of childbirth alone being the precipitating traumatic event. Good social support is a protective factor against the development of PTSD.^24^ In most cultural settings, there are postpartum practices in which experienced women stay with a new mother for a prescribed period of time, helping her adjust to her new role; this may confer coping skills and hence contribute to the low PTSD prevalence found in the current study.^25^ Evidence indicates that the expression of PTSD symptoms may vary due to differences in culture and that idioms of distress may influence the clinical presentation.^8^ Most studies investigating postpartum PTSD have been in Western countries. There is a need for the use of culturally appropriate measures to be used in further studies in our setting. There may have been an under or over-detection of PTSD as the measure used in this study has not been validated and may not be culturally appropriate. Our study observed a correlation between re-experiencing and avoidance symptoms, which is similar to other studies and consistent with the two-actor solution of re-experience or avoidance.^26^ The present study indicates that women who perceive childbirth as traumatic are more likely to go on to develop intrusion and avoidance symptoms. This highlights the need for further research in this field so as to offer targeted interventions.

### Study limitations

The lack of validation of the CBTS tool in the South African setting is a limitation of the current study. The design of the study prevents analysis of the prevalence of postpartum PTSD. The study sample was homogenous (single black African women) from a similar setting in Johannesburg, so generalisation of the findings should be made with caution. The sample size was small, thus limiting our generalisability and ability to explore the potential associated factors. Also, the pre-existing maternal PTSD, as well as trauma in the past 6 weeks, was not controlled for; hence, we are unable to conclude if the symptoms indicate new-onset PTSD. The sample only included data from women who had live births coming for follow-up in clinics (6 weeks postpartum); this may explain the lower percentage who met full diagnostic criteria for PTSD. The convenient sampling method used in the current study means that we should interpret the results of the study with caution, especially because some participants with the specific variables of interest may have been skipped, such as those with delayed onset of PTSD.

## Conclusion

The percentage of participants who met full diagnostic criteria for PTSD at 6 weeks postpartum was lower than expected compared to international research. However, the percentage of participants who perceived birth as a traumatic experience was in keeping with findings from international literature. This suggests the need for further research in the longer-term follow-up of women reporting birth as a traumatic experience but not experiencing full diagnostic PTSD symptoms at 6 weeks postpartum. There is a gap in knowledge on childbirth-induced PTSD and postpartum PTSD in South Africa and Africa generally. The current study has been beneficial in providing information on an under-investigated topic. Further study in this area is needed to assist in developing targeted policies and screening tools.
